# Inoculation against Mumps

**Published:** 1915-10

**Authors:** 


**Inoculation Against Mumps.** A. F. Hess, of New York, Arch. of Paed., August, inoculated 20 children using 6 to 8 c.c. of blood taken from the vein of the elbow and injecting intramuscularly. In some cases, the donor was barely convalescent, in others convalescent about 10 days, in others several years after an attack. The inoculated children were deliberately exposed but did not contract the disease, although controls did. Note—Here is a good illustration of human experimentation as charged by antivivisectionists against the medical profession. Mumps being semel-incident, almost inevitable and without a recorded death in an uncomplicated case (Furrell's Tyson's Practice) we consider the experiment justified, especially as there is no known germ from which to develope sterilized vaccines. But, we also feel that human experimentation should receive definite professional recognition, that carefully considered principles of its limitation should be laid down and that such experimentation should be controlled, either officially, or at least by some representative professional organization.

